# New-onset mental illness and long-term survival in survivors of critical illness: population-based cohort study in South Korea

**DOI:** 10.1192/bjo.2024.8

**Published:** 2024-03-22

**Authors:** Tak Kyu Oh, Hye Yoon Park, In-Ae Song

**Affiliations:** Department of Anesthesiology and Pain Medicine, Seoul National University Bundang Hospital, Seongnam, South Korea;; Department of Anesthesiology and Pain Medicine, College of Medicine, Seoul National University, Seoul, South Korea; Department of Psychiatry, Seoul National University Hospital, Seoul, South Korea

**Keywords:** Depressive disorders, epidemiology, mortality, outcome studies, primary care

## Abstract

**Background:**

Critical care unit (CCU) survivors have a high risk of developing mental illness.

**Aims:**

We aimed to examine the incidence and associated factors of newly developed mental illness among CCU survivors of critical illness. Moreover, we examined the association between newly developed mental illness and 2-year all-cause mortality.

**Method:**

All adult patients (≥20 years) who were admitted to the CCU during hospitalisation between 2010 and 2018 and survived for 1 year were defined as CCU survivors and were included in this nationwide population-based cohort study. CCU survivors with a history of mental illness before CCU admission were excluded from the study.

**Results:**

A total of 1 353 722 CCU survivors were included in the analysis; of these, 33 743 survivors (2.5%) had newly developed mental illness within 1 year of CCU admission. Old age, longer CCU stay, hospital admission through the emergency room, increased total cost of hospitalisation, mechanical ventilatory support, extracorporeal membrane oxygenation support and continuous renal replacement therapy were associated with an increased incidence of newly developed mental illness. Moreover, the newly developed mental illness group showed a 2.36-fold higher 2-year all-cause mortality rate than the no mental illness group (hazard ratio: 2.36; 95% CI: 2.30–2.42; *P* < 0.001).

**Conclusions:**

In South Korea, 2.5% of CCU survivors had newly developed mental illness within 1 year of CCU admission. Moreover, newly developed mental illness was associated with an increased 2-year all-cause mortality.

With advancing modern medical technology and systems, the survival rate of patients receiving critical care unit (CCU) care has increased.^1^ Intensivists often focus on the short- and intermediate-term outcomes of patients receiving CCU therapy. Long-term consequences of CCU survivors have recently become a source of concern. Post-intensive care syndrome includes cognitive (brain), physical and emotional symptoms that begin with critical illness and persist for a long time after CCU discharge.^2–6^ Mental illnesses can develop after CCU care. CCU circumstances may be uncomfortable and may harm the psychiatric health of patients there.^5^ These patients are isolated from familiar caregivers such as family members, and experience various unfamiliar treatments in a short time.^7^ Equipment such as ventilators, venous access devices, continuous renal replacement therapy (CRRT) and extracorporeal membrane oxygenation (ECMO) may be necessary for sustaining the life of these patients; the procedures for their placement are invasive, uncomfortable and painful.^2,8,9^ Critically, illness itself could also affect a patient's mental health.^10^ A meta-analysis of 27 studies from 1970 to 2015 revealed that 34% of CCU survivors had anxiety symptoms 12–14 months following CCU hospital stays.^11^ Another systematic review with meta-analysis of 15 studies from 1966 to 2007 revealed that 22% of CCU survivors reported post-CCU post-traumatic stress disorder (PTSD) symptoms.^12^ Moreover, a cohort study in the UK reported that depressive symptoms in CCU survivors were associated with a high 2-year mortality,^13^ which might reflect worsened relative long-term outcome after their CCU discharge. However, information regarding the relationship between mental illness and relative long-term survival outcomes such as 2-year mortality is still lacking. Therefore, the purpose of this study was to investigate the incidence and associated variables for newly developed mental illness among CCU survivors. Furthermore, we investigated the link between newly developed mental illness and 2-year all-cause mortality.

## Method

### Ethical statement, design and setting

The Institutional Review Board (IRB) of Seoul National University Bundang Hospital approved this retrospective population-based cohort research (IRB permission number: X-2102-666-904). The National Health Insurance Service's Big Data Center (NHIS-2021-1-620) also approved data sharing for this project. Because data analyses were done retrospectively utilising anonymised data from the South Korean NHIS database, informed consent was not required.

## Transparency declaration

In-Ae Song affirms that the manuscript is an honest, accurate and transparent account of the study being reported; that no important aspects of the study have been omitted; and that any discrepancies from the study as planned (and, if relevant, registered) have been explained.

## Prior presentation

This manuscript was presented as an abstract during the 42nd International Symposium on Intensive Care & Emergency Medicine, held on 21–24 March 2023 in Brussels, Belgium.

### Data source

For data extraction, we utilised the NHIS database. NHIS, South Korea's unified national health insurance, stores and handles data on illness diagnoses and prescriptions for treatments and/or medications. To obtain information on treatment expenses from the government, physicians (from all out-patient clinics and hospitals) must record all prescription information on procedures, drugs and illness diagnoses in the NHIS database. Disease diagnoses were recorded using the International Classification of Diseases, 10th revision (ICD-10 codes). Furthermore, the NHIS database provides demographic and socioeconomic status information for all patients in South Korea.

### Study population

All adults (aged 20 years or more) admitted to the CCU during their hospital stay between 2010 and 2018 and who lived for 1 year were classified as CCU survivors and included in this study. Data extraction used the prescription code for CCU admission during their stay. If a patient was admitted to the CCU twice or more throughout the research period, this study only included the first CCU admission case. Because their physical state in the CCU may have been poorer on the latter date of CCU admission than on the earlier date, this exclusion criterion strengthened the homogeneity of our study population. CCU survivors having a history of mental illness prior to CCU admission were excluded from this study to focus on newly diagnosed mental illness among CCU survivors.

The following legislative conditions must be met by CCU in South Korea. First, emergency resuscitation equipment, intubation devices, mechanical ventilators, defibrillators, electrocardiograms and respiratory function monitoring devices must always be available. Second, they must have a designated area that is larger than the standard ward, with at least 10 m^2^ per patient. Finally, for every two patients, they should have a committed doctor and at least one nurse. Fourth, the CCU should be powered by an uninterruptible power supply.

### Study endpoints

The primary endpoint in the present study was 2-year all-cause mortality (additional 1-year all-cause mortality) after CCU discharge. The death dates until 20 April 2021 were extracted from the NHIS Big Data Center on 20 April 2021 and used to calculate the survival time in this study. Because our study focused on 2-year all-cause mortality among CCU survivors in hospital till 31 December 2018, a 2-year follow-up period was considered sufficient. The secondary endpoint was newly diagnosed mental illness within 1 year from the date of CCU admission.

### Newly developed mental illness

Based on a previous study,^14^ we classified psychiatric disorders into four groups: schizophrenia spectrum disorders, mood disorders, anxiety disorders and other psychiatric disorders. The ICD-10 codes of the psychiatric disorders are presented in supplementary File 1 available at https://doi.org/10.1192/bjo.2024.8. Newly developed mental illness was evaluated for 1 year from the date of CCU admission among CCU survivors. CCU survivors who were diagnosed with newly developed mental illness comprised the newly developed mental illness group, while other patients comprised the no mental illness group. In the newly developed mental illness group, CCU survivors who were diagnosed with multiple (≥2) new mental illnesses comprised the newly developed multiple mental illnesses group.

### Covariates

Data pertaining to demographic and socioeconomic characteristics were extracted, including place of residence at the time of hospital admission, employment status, age and gender. The NHIS utilises the household income levels of individuals as a determinant of insurance premiums throughout the year. Government subsidies account for approximately 67% of medical expenses.^15^ Conversely, members of the medical aid programme, through which the government covers almost all medical expenses in an effort to alleviate the financial burden of medical costs, require enrolment. On the basis of quartile ratios, we categorised the patients into five distinct categories (Q1–Q4 and the medical aid programme group). The residential areas were categorised as rural (areas outside of Seoul and other major cities) or urban. Additionally, the duration of the hospital stays and care unit stays was documented. We classified the admitting departments into internal medicine or non-internal medicine departments. Additionally, patients’ admission to the CCU via the emergency room was documented. We considered individuals who underwent surgery while in hospital as having admission to the hospital associated with the surgery. There were three distinct hospital categories into which patients were admitted: tertiary general hospitals, general hospitals and other hospitals. In order to ascertain patients’ comorbidity status, ICD-10 codes were utilised to compute the Charlson comorbidity index (CCI) (supplementary File 2). CRRT, ECMO and mechanical ventilation support utilisation were monitored throughout the CCU stay. We categorised the subsequent occurrences into four groups: (a) follow-up at the same hospital, (b) transfer to a long-term facility care centre, (c) mortality occurring while in-patients, and (d) discharge and additional follow-ups at out-patient clinics. In addition, the dates of deaths that occurred during hospitalisation and discharge were documented. Additionally, we gathered the complete cost of hospitalisation (in USD) information. Using ICD-10 codes, the primary diagnosis at CCU admission was identified. The NHIS ascertained the primary diagnosis (a medical condition necessitating extensive examination or treatment throughout their hospital stay) for each person after their hospital discharge, or their demise.

### Statistical analysis

Continuous variables are represented as median values with interquartile ranges, whereas categorical variables are expressed as numbers accompanied by percentages. We compared clinicopathological characteristics of the newly developed mental illness group with those of the control group using the Mann–Whitney test for continuous variables and the chi-square test for categorical variables, respectively. To identify the factors associated with newly developed mental illness among CCU survivors, we subsequently fitted a multivariable logistic regression model to this data. The model for multivariable adjustment incorporated all covariates; the outcomes were displayed as odds ratios accompanied by 95% CIs. Using the Hosmer–Lemeshow test, we determined that the goodness of fit was adequate. To investigate the potential association between newly developed mental illness and 2-year all-cause mortality among CCU survivors, we fitted a multivariable Cox regression model for survival analysis, using 2-year all-cause mortality. A total of three distinct multivariable models were developed. Model 1 compared the 2-year all-cause mortality of individuals with newly developed mental illness with that of those without mental illness. In model 2, we compared the 2-year all-cause mortality rates in the newly developed single and multiple mental illnesses group with the no mental illness group. Model 3 compared the 2-year all-cause mortality of CCU survivors with schizophrenia spectrum disorders, mood disorders, anxiety disorders and other psychiatric illnesses, with that of the group of people without mental illness, taking into account the newly developed mental illness in detail. The models for multivariable adjustment incorporated all covariates, and the outcomes were displayed as hazard ratios accompanied by 95% CIs. The satisfaction of the central assumption of the Cox proportional hazards model was verified by using log-log plots. In all models, multicollinearity between variables was not an issue, as determined by the criterion of variance inflation factors being less than 2.0. We used R software (version 4.0.3; R Foundation for Statistical Computing, Vienna, Austria) to conduct each statistical analysis. We regarded *P* < 0.05 with two tails as a threshold for statistical significance.

## Results

### Study population

**Fig. 1:**
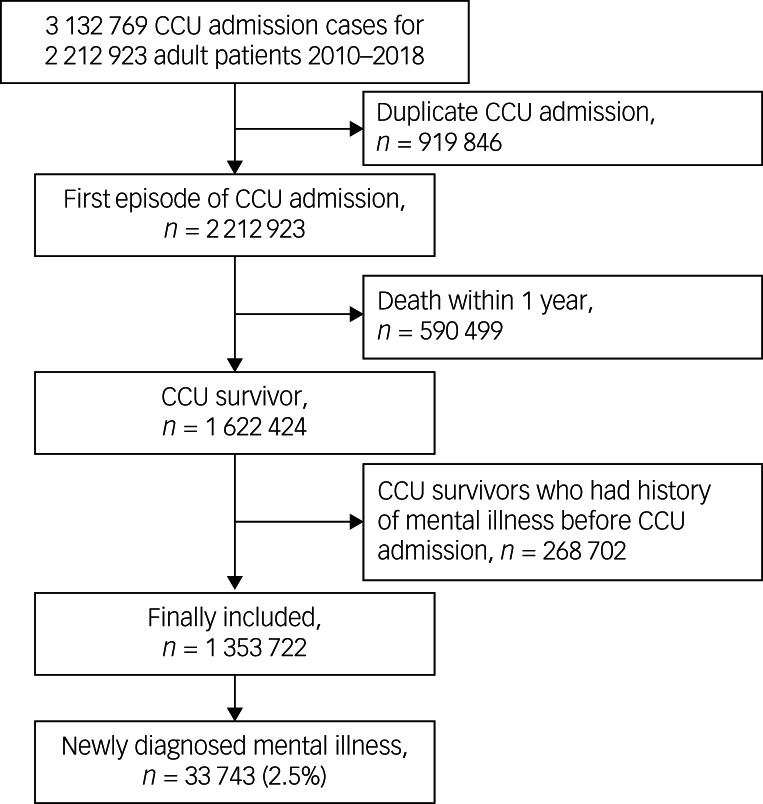
Flowchart depicting patient selection process for critical care unit (CCU).

Fig. 1 shows a flow chart depicting the patient selection process. There were 3 132 769 CCU admission cases of 2 212 923 adult patients between January 2010 and December 2018. Of 919 846 patients who were admitted to the CCU ≥ twice during the study period, their second and subsequent CCU admissions were excluded to focus only on their first CCU admission. After excluding 590 499 patients who died within 1 year of CCU admission, 1 622 424 CCU survivors were initially screened. Next, we excluded from the study 268 702 CCU survivors with a history of mental illness before CCU admission. Finally, we included in the analysis 1 353 722 CCU survivors, and found 33 743 survivors (2.5%) to have newly developed mental illness within 1 year of CCU admission. Table 1 shows the clinicopathological characteristics of CCU survivors. Two-year all-cause mortality occurred in 96 210 (7.1%) patients.

**Table 1:** Clinicopathological characteristics of CCU survivors

Variable	Median [IQR] or number (%) of total patients
Age, in years	65.0 [54.0, 76.0]
Gender, male	783 748 (57.9)
Having a job	731 312 (54.0)
Household income level	
Medical aid programme	108 554 (8.0)
Q1 (lowest)	235 840 (17.4)
Q2	229 175 (16.9)
Q3	291 630 (21.5)
Q4 (highest)	429 547 (31.7)
Unknown	58 976 (4.4)
Residence	
Urban area	543 207 (40.1)
Rural area	778 492 (57.5)
Unknown	32 023 (2.4)
CCU stay, in days	1.0 [1.0, 3.0]
LOS, in days	10.0 [6.0, 18.0]
CCI, point	2.0 [1.0, 3.0]
Admitting department	
Non-internal medicine	751 983 (55.5)
Internal medicine	601 739 (44.5)
Hospital admission through the emergency room	722 253 (53.4)
Type of hospital	
Tertiary general hospital	620 989 (45.9)
General hospital	708 212 (52.3)
Other hospital	24 521 (1.8)
Surgery-associated hospital admission	977 397 (72.2)
Mechanical ventilator support	206 635 (15.3)
ECMO support	2959 (0.2)
CRRT use	12 303 (0.9)
Result of hospitalisation	
Same hospital follow-up	312 063 (23.1)
Transfer to long-term care facility	48 752 (3.6)
Discharge, and other out-patient clinic follow-up	992 907 (73.3)
Total cost for hospitalisation, in USD	5629.7 [2,925.8, 8,976.3]
Newly developed mental illness	33 743 (2.5)
Newly developed single mental illness	28 182 (2.1)
Newly developed multiple mental illness	5561 (0.4)
Newly developed mental illness in detail	
Schizophrenia spectrum disorders	2562 (0.2)
Mood disorder	16 877 (1.2)
Anxiety disorder	13 713 (1.0)
Other psychiatric illness	6746 (0.5)
2-year all-cause mortality	96 210 (7.1)
Year of admission	
2010	163 553 (12.1)
2011	150 665 (11.1)
2012	147 315 (10.9)
2013	145 286 (10.7)
2014	147 883 (10.9)
2015	146 003 (10.8)
2016	153 150 (11.3)
2017	152 492 (11.3)
2018	147 375 (10.9)

IQR, interquartile range; CCU, critical care unit; LOS, length of hospital stays; CCI, Charlson comorbidity index; ECMO, extracorporeal membrane oxygenation; CRRT, continuous renal replacement therapy; USD, US dollars

### Associated factors for new-onset mental illness

Table 2 shows the results of the comparison between the newly developed mental illness and no mental illness groups. Table 3 shows the multivariable logistic regression model for newly developed mental illnesses among CCU survivors. Old age (odds ratio: 1.01; 95% CI: 1.01–1.01; *P* < 0.001), longer CCU stay (odds ratio: 1.03; 95% CI: 1.03–1.04; *P* < 0.001), hospital admission through emergency room (odds ratio: 1.17; 95% CI: 1.15–1.20; *P* < 0.001), increased total cost of hospitalisation (odds ratio: 1.01; 95% CI: 1.01–1.01; *P* < 0.001), mechanical ventilatory support (odds ratio: 1.71; 95% CI: 1.66–1.77; *P* < 0.001), ECMO support (odds ratio: 1.46; 95% CI: 1.25–1.71; *P* < 0.001) and CRRT use (odds ratio: 1.71; 95% CI: 1.58–1.86; *P* < 0.001) were associated with increased incidence of newly developed mental illness among CCU survivors.

**Table 2:** Comparison between the newly developed mental illness and no mental illness groups

Variable	Newly developed mental illness group, *n* = 33 743 median [IQR] or number (%)	No mental illness group, *n* = 1 319 979 median [IQR] or number (%)	*P*-value
Age, in years	69.0 [56.0, 78.0]	65.0 [54.0, 75.0]	<0.001
Gender, male	19 597 (58.1)	764 151 (57.9)	0.494
Having a job	13 841 (41.0)	717 471 (54.4)	<0.001
Household income level			<0.001
Medical aid programme	4004 (11.9)	104 550 (7.9)	
Q1 (lowest)	4865 (14.4)	230 975 (17.5)	
Q2	4469 (13.2)	224 706 (17.0)	
Q3	5571 (16.5)	286 059 (21.7)	
Q4 (highest)	8405 (24.9)	421 142 (31.9)	
Unknown	6429 (19.1)	52 547 (4.0)	
Residence			<0.001
Urban area	10 976 (32.5)	532 231 (40.3)	
Rural area	16 867 (50.0)	761 625 (57.7)	
Unknown	5900 (17.5)	26 123 (2.0)	
CCU stay, in days	2.0 [1.0, 5.0]	1.0 [1.0, 3.0]	<0.001
LOS, in days	10.0 [5.0, 20.0]	10.0 [6.0, 18.0]	<0.001
CCI, point	2.0 [1.0, 3.0]	2.0 [1.0, 3.0]	<0.001
Admitting department			<0.001
Non-internal medicine	17 547 (52.0)	734 436 (55.6)	
Internal medicine	16 196 (48.0)	585 543 (44.4)	
Hospital admission through the emergency room	21 004 (62.2)	701 249 (53.1)	<0.001
Type of hospital			<0.001
Tertiary general hospital	11 066 (32.8)	609 923 (46.2)	
General hospital	21 690 (64.3)	686 522 (52.0)	
Other hospital	987 (2.9)	23 534 (1.8)	
Surgery associated hospital admission	20 893 (61.9)	956 504 (72.5)	<0.001
Mechanical ventilator support	8439 (25.0)	198 196 (15.0)	<0.001
ECMO support	213 (0.6)	2746 (0.2)	<0.001
CRRT use	783 (2.3)	11 520 (0.9)	<0.001
Result of hospitalisation			<0.001
Same hospital follow-up	13 357 (39.6)	298 706 (22.6)	
Transfer to long-term care facility	2558 (7.6)	46 194 (3.5)	
Discharge, and other out-patient clinic follow-up	17 828 (52.8)	975 079 (73.9)	
Total cost for hospitalisation, USD	4,864.5 [2,206.5, 9,450.6]	5,644.0 [2,949.4, 8,967.9]	<0.001
Year of admission			<0.001
2010	3699 (11.0)	159 854 (12.1)	
2011	3545 (10.5)	147 120 (11.1)	
2012	3793 (11.2)	143 522 (10.9)	
2013	3765 (11.2)	141 521 (10.7)	
2014	3727 (11.0)	144 156 (10.9)	
2015	3857 (11.4)	142 146 (10.8)	
2016	3903 (11.6)	149 247 (11.3)	
2017	3823 (11.3)	148 669 (11.3)	
2018	3631 (10.8)	143 744 (10.9)	

The Mann–Whitney test for continuous variables and the chi-square test for categorical variables were used.

CCU, critical care unit; LOS, length of hospital stays; CCI, Charlson comorbidity index; ECMO, extracorporeal membrane oxygenation; CRRT, continuous renal replacement therapy; USD, US dollars

**Table 3:** Multivariable logistic regression model for newly developed mental illnesses among CCU survivors

Variable	Odds ratio (95% CI)	*P*-value
Age, in years	1.01 (1.01, 1.01)	<0.001
Gender, male	1.10 (1.08, 1.13)	<0.001
Having a job	0.92 (0.89, 0.94)	<0.001
Household income level		
Medical aid programme	1	
Q1 (lowest)	0.67 (0.64, 0.71)	<0.001
Q2	0.65 (0.62, 0.68)	<0.001
Q3	0.64 (0.61, 0.66)	<0.001
Q4 (highest)	0.66 (0.63, 0.69)	<0.001
Unknown	0.82 (0.75, 0.88)	<0.001
Residence		
Urban area	1	
Rural area	0.98 (0.95, 1.00)	0.070
Unknown	5.81 (5.38, 6.278)	<0.001
CCU stay, in days	1.03 (1.03, 1.04)	<0.001
CCI, point	1.01 (0.96, 1.02)	0.251
Admitting department		
Non-internal medicine	1	
Internal medicine	1.15 (1.13, 1.18)	<0.001
Hospital admission through the emergency room	1.17 (1.15, 1.20)	<0.001
Type of hospital		
Tertiary general hospital	1	
General hospital	1.66 (1.62, 1.70)	<0.001
Other hospital	1.80 (1.67, 1.93)	<0.001
Surgery associated hospital admission	0.66 (0.65, 0.68)	<0.001
Mechanical ventilator support	1.71 (1.66, 1.77)	<0.001
ECMO support	1.46 (1.25, 1.71)	<0.001
CRRT use	1.71 (1.58, 1.86)	<0.001
Result of hospitalisation		
Same hospital follow-up	1	
Transfer to long-term care facility	1.02 (0.97, 1.06)	0.526
Discharge, and other out-patient clinic follow-up	0.49 (0.48, 0.50)	<0.001
Total cost for hospitalisation, 1000 USD	1.01 (1.01, 1.01)	<0.001
Year of admission		
2010	1	
2011	1.08 (1.03, 1.14)	0.001
2012	1.20 (1.15, 1.26)	<0.001
2013	1.24 (1.18, 1.30)	<0.001
2014	1.19 (1.14, 1.25)	<0.001
2015	1.21 (1.16, 1.27)	<0.001
2016	1.19 (1.14, 1.25)	<0.001
2017	1.11 (1.05, 1.16)	<0.001
2018	1.13 (1.07, 1.18)	<0.001

CCU, critical care unit; LOS, length of hospital stays; CCI, Charlson comorbidity index; ECMO, extracorporeal membrane oxygenation; CRRT, continuous renal replacement therapy; USD, US dollars.

### Survival analyses

Table 4 shows the results of the multivariable Cox regression model for 2-year all-cause mortality among CCU survivors. In multivariable model 1, the group of people with newly developed mental illness showed a 2.36-fold higher 2-year all-cause mortality than the no mental illness group (hazard ratio: 2.36; 95% CI: 2.30–2.42; *P* < 0.001). All hazard ratios with 95% CIs for 2-year all-cause mortality of other covariates in multivariable model 1 are presented in supplementary File 3. In multivariable model 2, the newly developed single mental illness group and the newly developed multiple mental illnesses group showed 2.33-fold (hazard ratio: 2.33; 95% CI: 2.26–2.40; *P* < 0.001) and 2.50-fold (hazard ratio: 2.50; 95% CI: 2.36–2.65; *P* < 0.001) higher 2-year all-cause mortality, respectively, than the no mental illness group. In multivariable model 3, compared with the no mental illness group, CCU survivors with schizophrenia spectrum disorders (hazard ratio: 1.26; 95% CI: 1.15–1.38; *P* < 0.001), mood disorders (hazard ratio: 1.57; 95% CI: 1.51–1.63; *P* < 0.001), anxiety disorders (hazard ratio: 2.23; 95% CI: 2.15–2.32; *P* < 0.001) and other psychiatric illnesses (hazard ratio: 1.86; 95% CI: 1.76–1.96; *P* < 0.001) showed higher 2-year all-cause mortality.

**Table 4:** Multivariable Cox regression model for 2-year all-cause mortality among CCU survivors

Variable	Hazard ratio (95% CI)	*P*-value
Multivariable model 1		
No mental illness group	1	
Newly developed mental illness group	2.36 (2.30, 2.42)	<0.001
Multivariable model 2		
No mental illness group	1	
Newly developed single mental illness group	2.33 (2.26, 2.40)	<0.001
Newly developed multiple mental illness group	2.50 (2.36, 2.65)	<0.001
Multivariable model 3		
Newly developed mental illness in detail		
No mental illness group	1	
Schizophrenia spectrum disorders	1.26 (1.15, 1.38)	<0.001
Mood disorder	1.57 (1.51, 1.63)	<0.001
Anxiety disorder	2.23 (2.15, 2.32)	<0.001
Other psychiatric illness	1.86 (1.76, 1.96)	<0.001

All covariates that were included for multivariable adjustment are presented in supplementary file 2; CCU, critical care unit.

## Discussion

In this population-based cohort study, 2.5% of CCU survivors had newly developed mental illness within 1 year of CCU admission. Old age, longer CCU stay, hospital admission through the emergency room, increased total cost of hospitalisation, mechanical ventilatory support, ECMO support and CRRT use were potential risk factors for newly developed mental illness among CCU survivors. In addition, newly developed mental illness was associated with an increased 2-year all-cause mortality. To the best of our knowledge, this study is the first to show that newly developed mental illness among CCU survivors of critical illness who had no history of mental illness prior to CCU admission might have a poor long-term survival outcome.

From a nationally representative survey on mental illness conducted between 19 July 2011 and 16 November 2011, the lifetime prevalence of mental illness in South Korea was reported to be 27.6%;^16^ however, that study cannot be directly compared with our study, because our results show the incidence of mental illness in CCU survivors, as we excluded individuals with a history of any mental illness before CCU admission. For example, the reported incidence of schizophrenia spectrum disorders from 2008 to 2017 in South Korea was 0.05%,^17^ whereas in this study the incidence of schizophrenia spectrum disorders among CCU survivors was 0.2%. As there is no accurate information regarding the incidence of mental illnesses among both the general adult population and CCU survivors, further studies are warranted.

Few studies have shown that newly developed mental illnesses after CCU discharge are associated with high mortality rates. Mental illness is a major risk factor for mortality.^18^ A meta-analysis by Walker et al. suggested that the pooled relative risk of mortality among those with mental disorders was 2.22 (95% CI: 2.12–2.33).^18^ A study using postal questionnaires following discharge from the CCU in the UK revealed that in the first 2 years following discharge from the CCU, patients with depression symptoms had a 47% risk of mortality and were more likely to die than patients without depression symptoms.^13^ The major limitation was that the postal questionnaire study was based on a low response rate (36%).^13^ Although anxiety and PTSD symptoms were not associated with low survival rates in the UK postal survey, our study on various mental illnesses showed that newly developed mental illness was associated with increased risk of 2-year all-cause mortality.

Several studies have demonstrated that CCU survivors tend to be diagnosed with new-onset mental illnesses after discharge.^5,8,10,19^ CCU care was associated with an increased incidence of mood, anxiety and personality disorders and substance use, in comparison with both the in-patient cohort and general cohort over a 5-year period.^9,10^ Depression and anxiety symptoms occurred in a third of CCU survivors and persisted during a 12-month follow-up in a meta-analysis.^19^ CCU survivors tend to be diagnosed with PTSD, which negatively impacts their health-related quality of life.^5,20–22^ In contrast, a nationwide cohort study in Taiwan showed that people admitted to the CCU and general wards were associated with a higher risk of developing a mental disorder than the general population.^8^ Similarly, a 10-year population-based study conducted on in-patient adults (with no previous diagnosis of mental illness) in Ontario, Canada revealed that 121 101 CCU survivors were associated with a marginally increased risk of mental illness diagnosis compared with hospitalised patients without CCU care.^9^ Moreover, that study revealed that longer CCU stays or mechanical ventilatory support increased the risk of developing mental illness,^9^ which aligns with our results.

Risk factors for newly developed mental illness in CCU survivors in the present study were old age, no job status, poor economic status, admission to the internal medicine department, hospital admission through the emergency room, no surgery-associated admission (versus surgery-associated admission), general or other hospitals (versus tertiary general hospital), longer CCU stay, higher cost of hospital stay and invasive treatment in the CCU (mechanical ventilation, CRRT and ECMO support). Similar results have been reported in other studies.^9,10,20,21^ In the Ontario-Canadian population-based study, the adjusted hazard ratio of new mental illness diagnosis among CCU survivors was found to be lower in higher income groups than the lowest group (Quintile 1) and high in the female group with CCU admission, longer length of stay in the hospital and teaching hospital (versus small hospital), similar to our results.^9^ Canadian data found that mechanical ventilation seemed to be associated with a high risk of new-onset mental illness; however, unlike our data, renal replacement therapy was associated with a lower risk of newly diagnosed mental illness.^9^

The higher odds ratios of newly developed mental illness associated with mechanical ventilation, ECMO support and CRRT than newly developed mental illness associated with old age or length of CCU stay is an important finding. CCU patients who required invasive treatment such as mechanical ventilation, ECMO support and CRRT may have suffered from severe and critical illness at CCU admission. Additionally, invasive treatment such as mechanical ventilation, ECMO support and CRRT were also related to increased 2-year all-cause mortality among CCU survivors in this study. These findings suggest there may be a complex overlap of factors to explain the higher rate of 2-year all-cause mortality among those with newly developed mental illnesses. Therefore, rather than interpreting that a new mental illness alone increased long-term mortality, we should consider that the severity of the condition during the hospital stay as well as the after-effects of the treatment process all could have contributed to the increased 2-year all-cause mortality rate.

The impact of CCU admission on the development of mental illness could not be assessed in this study. This is because we did not compare the proportion of patients with new-onset mental illness between CCU survivors and other patients who were not admitted to the CCU. Sivanathan et al reported that CCU admission was associated with a marginally increased risk of mental illness diagnosis after a hospital stay, compared with in-patients without CCU admission.^23^ However, an understanding of the impact of CCU admission on mental illness diagnosis is still lacking, and more studies are needed on this issue.

The present study had some limitations. First, we could not approach individuals’ physical data such as body weight, height and nutritional status. Second, newly developed mental illness for 1 year after CCU admission could be an intermediate factor, with mortality being attributable to mechanical ventilatory support, ECMO support and CRRT during their hospital stay. However, we had to include intermediate factors in the multivariable Cox regression model, which could have created a bias in this study. Moreover, if new-onset mental illness is an intermediate variable, preventing mental illness might not show an estimated effect on death. Therefore, the association between new-onset mental illness and increased 2-year all-cause mortality might not be interpreted as causal. Third, there might be an immortal time bias because people who survived for 1 year were defined as CCU survivors and were included in this study. If CCU severity and length of CCU stay play a role in the onset of mental illness, then more severely ill patients are more likely to die within a year without developing a mental illness. Fourth, the Acute Physiology and Chronic Health Evaluation II score or simplified acute physiology score were not used to adjust the CCU data and disease severity of individuals during their CCU stay in this study. Fifth, examining newly developed illness within 1 year of CCU admission means that there was very likely a lost cohort of people who developed mental illness shortly after a year and whose illness was still related to CCU admission. This would be especially true for people whose CCU admission was prolonged and traumatic, with a high severity of physical illness. Lastly, the 2-year all-cause mortality follow-up period, including 2021, could have been influenced by the COVID-19 pandemic; we did not consider its impact on the study results.

In conclusion, 2.5% of CCU survivors in South Korea had newly developed mental illnesses within 1 year of CCU admission. Some factors were identified as potential risk factors for newly developed mental illness among CCU survivors. Moreover, newly developed mental illness was associated with an increased 2-year all-cause mortality.

## Supporting information

Oh et al. supplementary material 1 Oh et al. supplementary material

Oh et al. supplementary material 2 Oh et al. supplementary material

Oh et al. supplementary material 3 Oh et al. supplementary material

## Data Availability

The data that support the findings of this study are available from the corresponding author, I.-A.S., upon reasonable request.

## References

[1] Lerolle N, Trinquart L, Bornstain C, Tadié J-M, Imbert A, Diehl J-L, et al. Increased intensity of treatment and decreased mortality in elderly patients in an intensive care unit over a decade. Crit Care Med 2010; 38(1): 59–64. 19633539 10.1097/CCM.0b013e3181b088ec

[2] Inoue S, Hatakeyama J, Kondo Y, Hifumi T, Sakuramoto H, Kawasaki T, et al. Post-intensive care syndrome: its pathophysiology, prevention, and future directions. Acute Med Surg 2019; 6(3): 233–46. 31304024 10.1002/ams2.415 PMC6603316

[3] Jackson JC, Mitchell N, Hopkins RO. Cognitive functioning, mental health, and quality of life in ICU survivors: an overview. Crit Care Clin 2009; 25(3): 615–28. 19576534 10.1016/j.ccc.2009.04.005

[4] Jackson J, Ely EW, Morey MC, Anderson VM, Siebert C, Denne L, et al. Cognitive and physical rehabilitation of ICU survivors: results of the RETURN randomized, controlled pilot investigation. Crit Care Med 2012; 40(4): 1088. 22080631 10.1097/CCM.0b013e3182373115 PMC3755871

[5] Teixeira C, Rosa RG, Sganzerla D, Sanchez EC, Robinson CC, Dietrich C, et al. The burden of mental illness among survivors of critical care – risk factors and impact on quality of life: a multicenter prospective cohort study. Chest 2021; 160(1): 157–64. 33640377 10.1016/j.chest.2021.02.034

[6] Geense WW, van den Boogaard M, Peters MA, Simons KS, Ewalds E, Vermeulen H, et al. Physical, mental, and cognitive health status of ICU survivors before ICU admission: a cohort study. Crit Care Med 2020; 48(9): 1271. 32568858 10.1097/CCM.0000000000004443 PMC7434217

[7] Kamdar BB, Needham DM, Collop NA. Sleep deprivation in critical illness: its role in physical and psychological recovery. J Intensive Care Med 2012; 27(2): 97–111. 21220271 10.1177/0885066610394322 PMC3299928

[8] Peng F, Koh W-Y, Chung C-H, Chien W-C, Lin C-E. Risks of mental disorders among intensive care unit survivors: a nationwide cohort study in Taiwan. Gen Hosp Psychiatry 2022; 77: 147–54. 35660739 10.1016/j.genhosppsych.2022.05.007

[9] Sivanathan L, Wunsch H, Vigod S, Hill A, Pinto R, Scales DC. Mental illness after admission to an intensive care unit. Intensive Care Med 2019; 45(11): 1550–8. 31482222 10.1007/s00134-019-05752-5

[10] Sareen J, Olafson K, Kredentser MS, Bienvenu OJ, Blouw M, Bolton JM, et al. The 5-year incidence of mental disorders in a population-based ICU survivor cohort. Crit Care Med 2020; 48(8): e675–83. 32697508 10.1097/CCM.0000000000004413

[11] Nikayin S, Rabiee A, Hashem MD, Huang M, Bienvenu OJ, Turnbull AE, et al. Anxiety symptoms in survivors of critical illness: a systematic review and meta-analysis. Gen Hosp Psychiatry 2016; 43: 23–9. 27796253 10.1016/j.genhosppsych.2016.08.005 PMC5289740

[12] Davydow DS, Gifford JM, Desai SV, Needham DM, Bienvenu OJ. Posttraumatic stress disorder in general intensive care unit survivors: a systematic review. Gen Hosp Psychiatry 2008; 30(5): 421–34. 18774425 10.1016/j.genhosppsych.2008.05.006 PMC2572638

[13] Hatch R, Young D, Barber V, Griffiths J, Harrison DA, Watkinson P. Anxiety, depression and post traumatic stress disorder after critical illness: a UK-wide prospective cohort study. Crit Care 2018; 22(1): 1–13. 30466485 10.1186/s13054-018-2223-6 PMC6251214

[14] Nemani K, Li C, Olfson M, Blessing EM, Razavian N, Chen J, et al. Association of psychiatric disorders with mortality among patients with COVID-19. JAMA Psychiatry 2021; 78(4): 380–6. 33502436 10.1001/jamapsychiatry.2020.4442 PMC7841576

[15] Song YJ. The South Korean health care system. JMAJ 2009; 52(3): 206–9.

[16] Cho MJ, Seong SJ, Park JE, Chung IW, Lee YM, Bae A, et al. Prevalence and correlates of DSM-IV mental disorders in South Korean adults: the Korean epidemiologic catchment area study 2011. Psychiatry Invest 2015; 12(2): 164–70. 10.4306/pi.2015.12.2.164 PMC4390585 25866515

[17] Jung Y-S, Kim Y-E, Go D-S, Yoon S-J. The prevalence, incidence, and admission rate of diagnosed schizophrenia spectrum disorders in Korea, 2008–2017: a nationwide population-based study using claims big data analysis. PLoS ONE 2021; 16(8): e0256221. 34383865 10.1371/journal.pone.0256221 PMC8360527

[18] Walker ER, McGee RE, Druss BG. Mortality in mental disorders and global disease burden implications: a systematic review and meta-analysis. JAMA Psychiatry 2015; 72(4): 334–41. 25671328 10.1001/jamapsychiatry.2014.2502 PMC4461039

[19] Nikayin S, Rabiee A, Hashem MD, Huang M, Bienvenu OJ, Turnbull AE, et al. Anxiety symptoms in survivors of critical illness: a systematic review and meta-analysis. Gen Hosp Psychiatry 2016; 43: 23–9. 27796253 10.1016/j.genhosppsych.2016.08.005 PMC5289740

[20] Davydow DS, Gifford JM, Desai SV, Needham DM, Bienvenu OJ. Posttraumatic stress disorder in general intensive care unit survivors: a systematic review. Gen Hosp Psychiatry 2008; 30(5): 421–34. 18774425 10.1016/j.genhosppsych.2008.05.006 PMC2572638

[21] Asimakopoulou E, Madianos M. The prevalence of major depression–PTSD comorbidity among ICU survivors in five general hospitals of Athens: a cross-sectional study. Issues Ment Health Nurs 2014; 35(12): 954–63. 25325150 10.3109/01612840.2014.924609

[22] Dowdy DW, Eid MP, Sedrakyan A, Mendez-Tellez PA, Pronovost PJ, Herridge MS, et al. Quality of life in adult survivors of critical illness: a systematic review of the literature. Intensive Care Med 2005; 31(5): 611–20. 15803303 10.1007/s00134-005-2592-6

[23] Sivanathan L, Wunsch H, Vigod S, Hill A, Pinto R, Scales DC. Mental illness after admission to an intensive care unit. Intensive Care Med 2019; 45(11): 1550–8. 31482222 10.1007/s00134-019-05752-5

