# Supplementary material for: New-onset mental illness and long-term survival in survivors of critical illness: population-based cohort study in South Korea
Source: BJPsych Open. 2024 Mar 22;10(2):e70. doi: 10.1192/bjo.2024.8 (PMC10988599; doi:10.1192/bjo.2024.8)
Supplement: Oh et al. supplementary material 1 — Oh et al. supplementary material [file S2056472424000085sup001.docx]

***Additional file.** ***ICD-10* codes associated with each psychiatric diagnostic category**

| Schizophrenia spectrum disorders | F20* (schizophrenia)  F22* (delusional disorders)  F23* (acute and transient psychotic disorders)  F25* (schizoaffective disorder)  F28 (other nonorganic psychotic disorders)  F29 (unspecified nonorganic psychosis) |
| --- | --- |
| Mood disorders | F30* or F31* (bipolar disorder)  F32* or F33* (major depressive disorder)  F34* (persistent mood disorder)  F39* (unspecified mood disorder) |
| Anxiety disorders | F40* (phobic anxiety disorders) F41* (other anxiety disorders) |
| Other psychiatric disorders | Excludes diagnoses above and  F42* (Obsessive-compulsive disorder)  F43* (Reaction to severe stress, and adjustment disorders)  F44* (Dissociative and conversion disorders)  F45* (Somatoform disorders)  F48* (Other nonpsychotic mental disorders)  F50* (Eating disorders)  F51* (Sleep disorders not due to a substance or known physiological condition)  F53* (Mental and behavioral disorders associated with the puerperium)  F60* (Other specific personality disorders)  F68* (Other disorders of adult personality and behavior)  F69* (Unspecified disorder of adult personality and Behavior)  F90* (Attention-deficit hyperactivity disorders)  F95* (Tic disorder)  F99 (Mental disorder, not otherwise specified) |
