# Supplementary material for: New-onset mental illness and long-term survival in survivors of critical illness: population-based cohort study in South Korea
Source: BJPsych Open. 2024 Mar 22;10(2):e70. doi: 10.1192/bjo.2024.8 (PMC10988599; doi:10.1192/bjo.2024.8)
Supplement: Oh et al. supplementary material 3 — Oh et al. supplementary material [file S2056472424000085sup003.docx]

Additional File 3. All HRs with 95% CIs for 2-year all-cause mortality of other covariates in multivariable model 1

| Variable | | HR (95% CI) | *P*-value |
| --- | --- | --- | --- |
| Age, year | | 1.05 (1.05, 1.05) | <0.001 |
| Sex, male | | 1.31 (1.29, 1.33) | <0.001 |
| Having a job | | 0.96 (0.95, 0.97) | <0.001 |
| Household income level | |  |  |
|  | Medical aid program | 1 |  |
|  | Q1 (Lowest) | 0.79 (0.77, 0.81) | <0.001 |
|  | Q2 | 0.78 (0.76, 0.80) | <0.001 |
|  | Q3 | 0.75 (0.73, 0.76) | <0.001 |
|  | Q4 (Highest) | 0.72 (0.70, 0.74) | <0.001 |
|  | Unknown | 0.79 (0.75, 0.82) | <0.001 |
| Residence | |  |  |
|  | Urban area | 1 |  |
|  | Rural area | 1.03 (1.02, 1.05) | <0.001 |
|  | Unknown | 1.57 (1.49, 1.66) | <0.001 |
| CCU stay, day | | 1.03 (1.03, 1.04) | <0.001 |
| CCI, point | | 1.20 (1.20, 1.21) | <0.001 |
| Admitting department | |  |  |
|  | Non-IM | 1 |  |
|  | IM | 1.06 (1.05, 1.08) | <0.001 |
| Hospital admission through ER | | 1.04 (1.03, 1.06) | <0.001 |
| Type of hospital | |  |  |
|  | Tertiary general hospital | 1 |  |
|  | General hospital | 0.99 (0.98, 1.00) | 0.146 |
|  | Other hospital | 1.20 (1.16, 1.25) | <0.001 |
| Surgery associated hospital admission | | 0.80 (0.79, 0.81) | <0.001 |
| Mechanical ventilator support | | 1.24 (1.22, 1.27) | <0.001 |
| ECMO support | | 0.62 (0.42, 1.25) | 0.124 |
| CRRT use | | 1.07 (1.02, 1.13) | 0.010 |
| Result of hospitalization | |  |  |
|  | Same hospital follow up | 1 |  |
|  | Transfer to long-term care facility | 1.22 (1.18, 1.26) | <0.001 |
|  | Discharge, and other outpatient clinic follow up | 0.86 (0.84, 0.87) | <0.001 |
| Total cost for hospitalization, 1000 USD | | 1.01 (1.01, 1.01) | <0.001 |
| Year of admission | |  |  |
|  | 2010 | 1 |  |
|  | 2011 | 0.96 (0.94, 0.98) | 0.001 |
|  | 2012 | 0.90 (0.87, 0.91) | <0.001 |
|  | 2013 | 0.87 (0.85, 0.90) | <0.001 |
|  | 2014 | 0.81 (0.79, 0.83) | <0.001 |
|  | 2015 | 0.77 (0.75, 0.79) | <0.001 |
|  | 2016 | 0.75 (0.73, 0.77) | <0.001 |
|  | 2017 | 0.72 (0.70, 0.74) | <0.001 |
|  | 2018 | 0.70 (0.68, 0.72) | <0.001 |

HR, hazard ratio; CI, confidence interval; CCU, critical care unit; CCI, Charlson comorbidity index; IM, internal medicine; ECMO, extracorporeal membrane oxygenation; CRRT, continuous renal replacement therapy; USD, United States Dollars
